# Initial Experience of Sorafenib Neoadjuvant Therapy Combined with Retroperitoneoscopy in Treating T2 Large Renal Carcinoma

**DOI:** 10.1155/2015/609549

**Published:** 2015-09-03

**Authors:** Chun-hua Lin, He-jia Yuan, Ke Wang, Ji-tao Wu, Qing-zuo Liu, Sheng-qiang Yu, Chang-ping Men, Zhen-li Gao, Jiahui Wang

**Affiliations:** Department of Urology, Yantai Yuhuangding Hospital, Medical College of Qingdao University, 20 Yuhuangding East Road, Yantai, Shandong 264000, China

## Abstract

*Objectives*. To investigate the safety and feasibility of sorafenib neoadjuvant therapy combined with retroperitoneoscopic radical nephrectomy (RRN) in treating T2 large renal cell carcinoma (RCC). *Methods*. Retrospectively analyzed 5 cases (2 males and 3 females, aged 52–73 years) of T2 stage large RCC who receive preoperative sorafenib targeted treatment (400 mg bid for 1–3 months) and RRN between March, 2013, and July, 2014. Patient information, therapeutic regimen, drug adverse effect, tumor changes before and after surgery, and perioperative parameters were recorded. *Results*. During the sorafenib therapy adverse effects included 2 cases of hypertension (Grade I toxicity), 1 case of hand-foot syndrome (Grade I), and 1 case of diarrhea (Grade II), which were all tolerable for patients. CT scan and histopathological tests confirmed significant reduction in the longest dimension (LD) and medium density (MD) of the tumor after therapy as well as tumor hemorrhage, necrosis, and cystic degeneration. All 5 patients received RRN surgery successfully around 2 weeks after drug discontinuation with only 1 case of perioperative complication. *Conclusions*. Sorafenib neoadjuvant therapy could significantly reduce the size and aggressiveness of T2 large renal tumors, thus reducing the operative challenge and enabling patients who were previously disqualified for operation to receive surgical treatment.

## 1. Introduction

Renal tumor is the second most common carcinoma among urologic neoplasms with the highest fatality rate. It accounts for 2-3% of all malignancies and 80–90% of renal malignant tumors. The fatality rate is 30–40% comparing to the 20% of prostatic cancer or bladder cancer [1]. Radical nephrectomy is the main method for treating early stage renal tumors. However, it has limited use in treating metastatic renal cell carcinoma (RCC), for which molecular targeted drugs have huge advantages. According to multi-institutional studies worldwide, molecular targeted drug is effective against advanced renal tumor and increases the chance of advanced tumor patients receiving radical surgery or nephron-sparing surgery. Our hospital started using sorafenib as neoadjuvant targeted drug since 2013 in treating RCC patients with large tumors presumed not suitable for retroperitoneoscopic radical nephrectomy (RRN). Before 2013 RRN surgery on 3 patients with similar conditions had been performed but all failed in tumor removal. We hereby report our initial experience of successfully treating 5 cases of advanced RCC using preoperational sorafenib neoadjuvant therapy combined with RRN.

## 2. Materials and Methods

### 2.1. Patient Information

The clinical information of 5 renal tumor patients (2 males and 3 females; age 52–73, average of 66 years old), who voluntarily received sorafenib adjuvant therapy and RRN surgery between March, 2013, and July, 2014, was retrospectively analyzed. The inclusion criteria were patients diagnosed with RCC through needle biopsy, with tumor diameter > 7 cm, and CT scan showed large tumor with adhesion to surrounding tissues which was anticipated to be difficult for surgical removal or might cause large amount of intraoperative blood loss. The exclusion criteria were RCC with clear boundary from surrounding tissues which could be surgically removed. Patients' cardiopulmonary and coagulation functions were assessed before operation and no apparent surgical contraindications were identified.

### 2.2. Treatment

All 5 patients were treated according to international recommendations [2]. Sorafenib was continuously delivered orally at 400 mg bid for 36–81 d, with average delivery time of 51.4 d. During the drug treatment, the cellular evaluation and biochemical analysis of blood and the coagulation indices (TT, FIB, PT, and APTT) of patients were regularly monitored and their cardiopulmonary functions were thoroughly evaluated to exclude surgical contraindications. Histopathological tests were performed before and after the sorafenib treatment. An average of 11.6 days after discontinuation of sorafenib treatment, patients received RRN under endotracheal general anesthesia. Surgical specimens were sent for pathological analysis.

### 2.3. Statistics

CT urography test was performed on all patients prior to medication and operation. Response evaluation criteria in solid tumor (RECIST) [3] were adopted to evaluate the effect of sorafenib on tumor. The longest dimension (LD) of primary tumors was measured from 3-dimensional reconstructed images from thin layer CT scan (1.5 mm) using Image Workstation (Terra Recon, San Mateo, CA). The medium density (MD) of tumor was measured using the Choi standard [4] according to their enhancement degree. Drug toxicity was graded according to the common terminology criteria for adverse events (CTCAE, version 4.0). The patients' general information, treatment protocol, and perioperative parameters were recorded. Statistical analysis was performed using SPSS 19.0 software. Significance was calculated using *t*-test; *P* < 0.05 indicated statistically significant difference.

### 2.4. Statement of Human Rights

This experiment was conducted with the understanding and the consent of the human subject. All procedures were performed in agreement with the ethical standards of the Ethics Committee of Yantai Yuhuangding Hospital. Written informed consents were obtained from each patient for enrolling in this study.

## 3. Results

**Figure 1:**
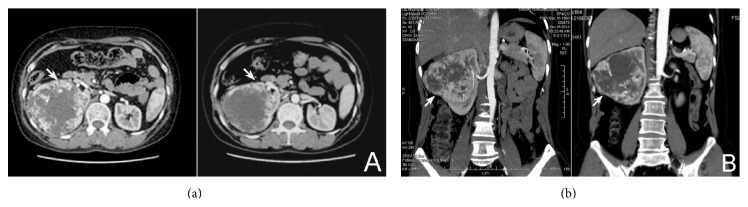
Transverse (a) and coronal (b) CT scans of a patient before (left) and after (right) sorafenib treatment. Arrows indicated the renal tumor.

**Figure 2:**
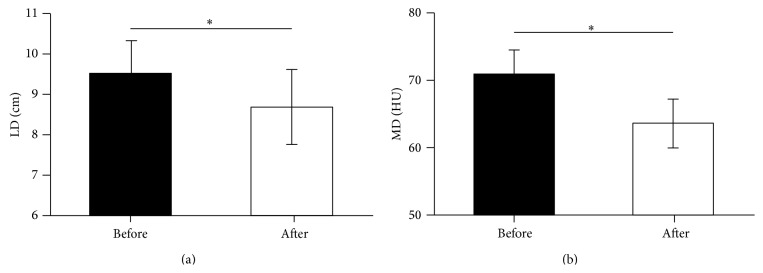
(a) Comparison of the longest dimension (LD) of tumors before and after sorafenib treatment. (b) Comparison of the medium density (MD) of tumors before and after sorafenib treatment. Error bars showed the standard error while ∗ represented significant difference (*P* < 0.05).

**Figure 3:**
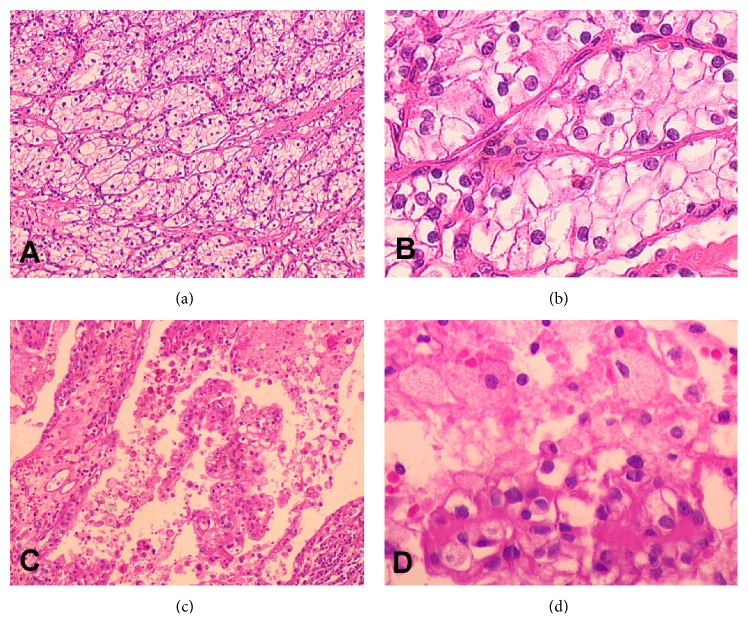
Histopathologic staining of RCC from the same patient before (a and b) and after (c and d) sorafenib treatment, where tumor hemorrhage, necrosis, and cystic degeneration were visible. (a) and (c) were at ×100 magnification. (b) and (d) were at ×400 magnification.

**Figure 4:**
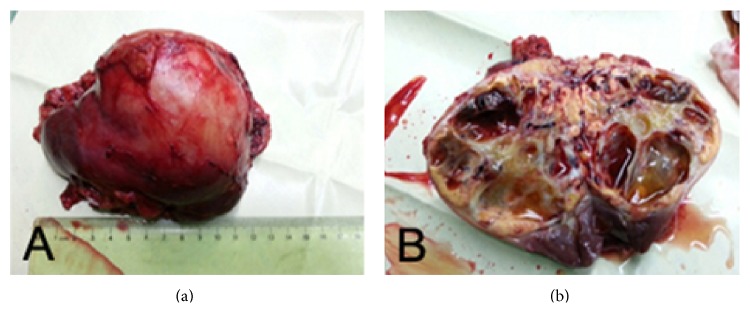
Image of a surgical extracted tumor (a) and its dissected view (b).

Patients' information, therapeutic outcome, and perioperative parameters were summarized in Table 1. During the course of drug treatment patient #1 had no particular discomfort (Grade 0). Patients #2, #3, and #4 experienced Grade I adverse event including mild hypertension (despite normal blood pressure before medication and no history of hypertension) and hand-foot syndrome with mild abnormal sensations one week after drug administration. None of them required medical intervention. Patient #5 had diarrhea 10 days after taking sorafenib. Taking the drug episodically with a 7-day gap relieved the symptom (Grade II). No other patient had dose reduction or gap of drug administration except patient #5. The adverse effects of sorafenib were tolerable. CTU test showed that in all 5 patients the tumor size reduced and enhancement receded in CT scan (Figure 1). The LD of primary tumors reduced significantly from 9.54 ± 1.85 cm to 8.72 ± 2.09 cm (*P* = 0.034) (Figure 2(a)) and the MD of tumors from 70.8 ± 8.26 HU to 63.6 ± 8.17 HU (*P* = 0.019) after sorafenib treatment (Figure 2(b)). Histopathologic test showed that after sorafenib treatment tumor hemorrhage, necrosis, and cystic degeneration were observed (Figure 3), indicating reduced aggressiveness of the tumor. Patients were successfully operated upon 7–16 days (average 11.6 days) after discontinuation of sorafenib. There were no severe perioperative complications except that patient #2 suffered a more severe adhesion of the tumor to the surrounding tissue than other patients. This resulted in prolonged operation, intraoperative hemorrhea, and damage in the peritoneum, which was immediately occluded with Hem-o-lok clip before the completion of the surgery. Surgical specimens were dissected, showing expanded lumen and apparent liquefaction (Figure 4). Pathological examination showed 4 cases were Grade II clear cell renal cell carcinoma (CCRCC) and one Grade I CCRCC. All patients were discharged within 2 weeks after the surgery.

## 4. Discussion

Despite the rising early stage diagnosis rate for renal tumors in recent years, there is still a large portion of patients who have missed the best time for operation upon diagnosis. Renal carcinoma tumors are highly resistant to radio- or chemotherapies for unknown reasons. Therefore palliative nephrectomy or immunotherapy is often used, such as IFN-*α* and IL-2. However, the response rate is as low as 15%. Currently, molecular sorafenib has been widely used in treating late-stage RCC. Escudier et al. carried out a phase III multicenter trial of sorafenib treatment in renal cancer which showed that patients treated with sorafenib had median PFS that was twice as long as the placebo group (5.5 versus 2.8 months) and their quality of life (QOF) was significantly higher [5]. Furthermore, Ye and Zhang reported that sorafenib was more effective in patients of Chinese ethnicity than in western patients and was well tolerated even at higher dosage and when used in combination with other anticancer agents [6].

There is controversy regarding the combination of molecular targeted therapy and retroperitoneoscopic surgery. It is currently understood that, for patients with localized advanced RCC, neoadjuvant therapy could be used first to reduce the tumor size, making the tumor easier for surgical resection or preserving renal functions as much as possible [7, 8]. For metastatic RCC, it is recommended to receive cytoreductive surgery before targeted treatment, the selection criteria of which are (A) ECOG score 0-1; (B) >75% of tumor load that could be resected; (C) no other obvious organ dysfunctions. On the other hand, for patients whose resection nidus comprises < 75% of the tumor load, neoadjuvant therapy is recommended [9]. In the application of RRN in resecting large tumors (>7 cm), the safety and effectiveness of the procedure, as well as the definition of the limit of tumor size, are still controversial. Albqami and Janetschek proposed that when tumor was confined within Gerota's fascia, RRN is applicable regardless of the size of the tumor. Many other researchers believed that, on the basis of good laparoscopic skills, RRN can be applied to all RCC confined in renal dispose capsule. Therefore the size of tumors is not the only standard to determine laparoscopy or open surgery.

Consensus has not been reached over the time window of preoperative sorafenib neoadjuvant treatment for RCC, the discontinuation time of the drug, and the time of performing the operation. There were large differences among various case reports. Most people believe that operation should only be considered after the primary or metastatic tumors had reduced by a certain extent and the tumor size should be continually monitored after discontinuation of sorafenib. If the tumor size increased again, it was not suitable for operation.

In this study, sorafenib treatment combined with retroperitoneoscopy was effective against RCC that was originally considered difficult to be surgically removed. In all 5 patients the renal tumors diminished to various extents. However, there is no unified criteria for evaluating the efficacy of sorafenib in treatment of primary RCC. We used the RECIST method to assess the changes of tumors after treatment. Histopathological changes could also reflect the necrosis of carcinoma, thus providing further evidence for using sorafenib in targeted therapy for RCC.

It has been reported that long-term use of sorafenib might severely delay the postoperative wound healing and increase the risk of postoperative bleeding and formation of thrombus. Kondo et al. reported it safe to stop sorafenib 7 days before operation [10] although many others suggested a 2-week gap between drug discontinuance and operation. In our study operations were performed 7–16 days after drug discontinuance. No obvious intraoperative or postoperative complications occurred except for #2 due to the heavy tumor adhesion. All 5 patients left hospital within 2 weeks after the operation.

At the moment, it is unknown whether sorafenib could reduce the recurrence risk of advanced RCC. According to the report by Cleveland Clinic Foundation, the positive surgical margin rates for laparoscopic removal of T1 stage (median LD 4.5 cm) and T2 stage (median LD 9.5 cm) RCC were close, with no difference in survival rates 2 years after operation [11].

This study is a retrospective study and relatively limited in the number of cases included. We did not assess the 5-year cancer-free survival rate or overall survival rate and, therefore, could not evaluate the long-term effect of this combined therapy. Larger sample size and longer follow-up period are required to further evaluate the clinical effect of sorafenib combined RPN in treating T2 large renal tumors.

In conclusion, neoadjuvant therapy based on multitargeted drugs plays an important part in comprehensive treatment of late-stage RCC. Sorafenib neoadjuvant therapy could contribute to reducing the size and aggressiveness of tumors, thus reducing the operative challenge and increasing the radical resection rate of late-stage carcinoma, enabling patients to receive surgical treatment who were previously disqualified for operation.

## Figures and Tables

**Table 1:** Patients' information and perioperative parameters of the sorafenib treatment group.

Patient	Gender	Age (yr)	TNM staging	Drug administration time (d)	Drug discontinuation time (d)	Drug side effects	Toxicity grading	LD before medication (cm)	LD after medication (cm)	MD before medication (HU)	MD after medication (HU)	Operation time (min)	Intraoperative blood loss (mL)	Perioperative complications	Fuhrman grading	Length of stay (d)
1	F	52	T2aN0M0	41	13	None	0	8.8	7.1	73	59	190	650	None	I	8
2	M	65	T2bN1M1	36	8	Hypertension	I	12.1	11.9	69	61	225	1980	Intraoperative hemorrhea and peritoneal damage	II	14
3	M	71	T2aN0M1	48	14	Hand-foot syndrome	I	9.1	8.2	82	77	210	530	None	II	11
4	F	69	T2aN1M1	51	16	Hypertension	I	10.5	9.6	59	56	102	810	None	II	13
5	F	73	T2aN0M0	81	7	Diarrhea	II	7.2	6.8	71	65	120	480	None	II	12

LD: longest dimension of the tumor; MD: medium density of the tumor.
